# SEABIRD: RECRUITING A DIVERSE COMMUNITY-BASED POPULATION FOR AN ALZHEIMER’S BLOOD TEST STUDY

**DOI:** 10.1093/geroni/igae098.4070

**Published:** 2024-12-31

**Authors:** Matthew Larsen, Melody Li, Melanie Burton, Lisa Soke, Rickey George, Randall Bateman

**Affiliations:** Washington University in St. Louis, St. Louis, Missouri, United States; Washington University in St. Louis, St. Louis, Missouri, United States; Washington University School of Medicine, St. Louis, Missouri, United States; Washington University School of Medicine, St. Louis, Missouri, United States; Washington University in St. Louis, St. Louis, Missouri, United States; Washington University in St. Louis, St. Louis, Missouri, United States

## Abstract

Older adults, often isolated and facing health challenges, are less integrated into society and research, making it crucial for Alzheimer’s disease (AD) studies to effectively recruit them. The Study to Evaluate Amyloid in Blood and Imaging Related to Dementia (SEABIRD) enrolled 1,122 participants to determine the participant acceptability and accuracy (relative to amyloid PET) of a plasma amyloid-β 42/40 assay in a diverse, community-based sample of older adults. Participants completed a blood collection, cognitive screening, and a survey about their study experience and perceptions of the blood test. A subset of participants completed additional blood collection for reproducibility, amyloid PET and MRI for validation of the blood test, and the Clinical Dementia Rating® for validation of the cognitive screening measures. To ensure a diverse sample of participants, demographic factors such as race and education were prioritized during the recruitment process. Participants were recruited through existing study registries, electronic medical record chart reviews, newspaper postings, flyers, and community events. Of the participants enrolled, 23.5% self-identified as Black or African American, exceeding the study’s goal of 14% for the US population and 5.2% for a large AD research cohort (the Alzheimer’s Disease Neuroimaging Initiative [ADNI] study in 2022). Additionally, SEABIRD participants were more likely to have a high school education or less (25.3%) compared to those in ADNI (15.5%). SEABIRD achieved broader diversity than typical AD cohorts and may help inform practices to recruit underrepresented populations. These results demonstrate the feasibility of recruiting a diverse, community-based population for AD blood collection studies.

